# Biosynthesis of spinel nickel ferrite nanowhiskers and their biomedical applications

**DOI:** 10.1038/s41598-021-96918-z

**Published:** 2021-08-31

**Authors:** Hajar Q. Alijani, Siavash Iravani, Shahram Pourseyedi, Masoud Torkzadeh-Mahani, Mahmood Barani, Mehrdad Khatami

**Affiliations:** 1 Department of Biotechnology, Shahid Bahonar University of Kerman, Kerman, Iran; 2 Research and Technology Institute of Plant Production (RTIPP), Shahid Bahonar University of Kerman, Kerman, Iran; 3 Faculty of Pharmacy and Pharmaceutical Sciences, Isfahan University of Medical Sciences, Isfahan, Iran; 4 Biotechnology Department, Institute of Science and High Technology and Environmental Sciences, Graduate University of Advanced Technology, Kerman, Iran; 5 Medical Mycology and Bacteriology Research Center, Kerman University of Medical Sciences, 7616913555 Kerman, Iran; 6 Noncommunicable Diseases Research Center, Bam University of Medical Sciences, Bam, Iran; 7 Department of Medical Biotechnology, Faculty of Medical Sciences, Tarbiat Modares University, Tehran, Iran; 8 Cell Therapy and Regenerative Medicine Comprehensive Center, Kerman University of Medical Sciences, Kerman, Iran

**Keywords:** Biochemistry, Biotechnology, Microbiology, Nanoscience and technology

## Abstract

Greener methods for the synthesis of various nanostructures with well-organized characteristics and biomedical applicability have demonstrated several advantages, including simplicity, low toxicity, cost-effectiveness, and eco-friendliness. Spinel nickel ferrite (NiFe_2_O_4_) nanowhiskers with rod-like structures were synthesized using a simple and green method; these nanostructures were evaluated by X-ray diffraction analysis, transmission electron microscopy, scanning electron microscopy, and X-ray energy diffraction spectroscopy. Additionally, the prepared nanowhiskers could significantly reduce the survival of *Leishmania major* promastigotes, at a concentration of 500 μg/mL; the survival of promastigotes was reduced to ≃ 26%. According to the results obtained from MTT test (in vitro), it can be proposed that further studies should be conducted to evaluate anti-leishmaniasis activity of these types of nanowhiskers in animal models.

## Introduction

The nanowhiskers with unique shape, electrical, optical, magnetic, and surface properties have shown attractive clinical and biomedical potentials^1,2^. Typically, the production of different nanostructures with well-organized morphologies and sizes is highly demanded by researchers and scientists due to their unique applications and properties^3–7^.

Inorganic nanostructures with different mechanical and physical properties can be employed in different applications such as medicine, electronic device, sunscreens, military applications, photovoltaic cells, paints, catalysts, and among others^8–16^. Among nanostructures, nanofibers are defined as structures with an outer diameter below 1000 nm^17,18^. Nanowhisker is a type of nanofiber crystal with a diameter of less than 100 nm^19^. Nanowhiskers can have various applications in filtration^20,21^, food packaging^22^, diagnosis^23^, drug delivery^24^, gene delivery^25–27^, cancer therapy^28^ and cell scaffolding^29^.

In recent years, the mechanical properties and widespread applications of NiFe_2_O_4_ nanowhiskers have been demonstrated by researchers. Ferrites are ceramics made from a combination of iron oxide and divalent metals such as barium, strontium, lead, nickel, cobalt, among others ^30,31^. Ferrites have wide applications in various biomedical ^32^, catalytic ^27,33–35^, wastewater ^36^, extraction ^37^, electrical ^38^ fields.

For the fabrication of nanowhiskers, various synthesis approaches have been reported, including microwave^39^, carbo-thermal reduction^40^, and electrospinning ^41^ techniques. However, one of the important drawbacks with these methods is the dependence on expensive equipment or energy consumption, which is directly or indirectly a threat to environmental health ^42^. To solve this problem, it is necessary to discover environmentally friendly production methods for the synthesis of nanostructures ^43–47^. Therefore, the green synthesis of nanostructures in various forms has been developed using plant extracts^48^. The application of plant extracts for synthesizing nanostructures is in accordance with the principles of green chemistry. This bio-based method has some important advantages of low toxicity and eco-friendliness, and for the creation of nanostructures, plant extracts act as natural reducing^49^ and stabilizing agents^50–52^.

As far as we know, no studies have been conducted for greener biosynthesis of spinel ferrite nanowhiskers; therefore, we focused on greener synthesis of spinel ferrite nanowhiskers using rosemary extract. Rosemary (*Rosmarinus officinalis*) is a woody, evergreen and fragrant medicinal plant, which contains phytochemicals such as rosmarinic acid, betulinic acid, camphor, carnosic acid, caffeic acid, carnosol, and ursolic acid. The active ingredients in rosemary have suitable antioxidant, anti-inflammatory, and antibacterial effects.

Aqueous extract of rosemary was utilized to make NiFe_2_O_4_ nanostructures in a single step at pH 7. X-ray powder diffraction (XRD), high-resolution transmission electron microscopy (HR-TEM), scanning electron microscopy (SEM), and energy-dispersive X-ray spectroscopy (EDX) techniques were employed to characterize these nanostructures. Additionally, antiparasitic activities of these nanostructures against *Leishmania major* were evaluated, in vitro.

## Results

### Characterization of NiFe_2_O_4_nanowhiskers

**Figure 1:**
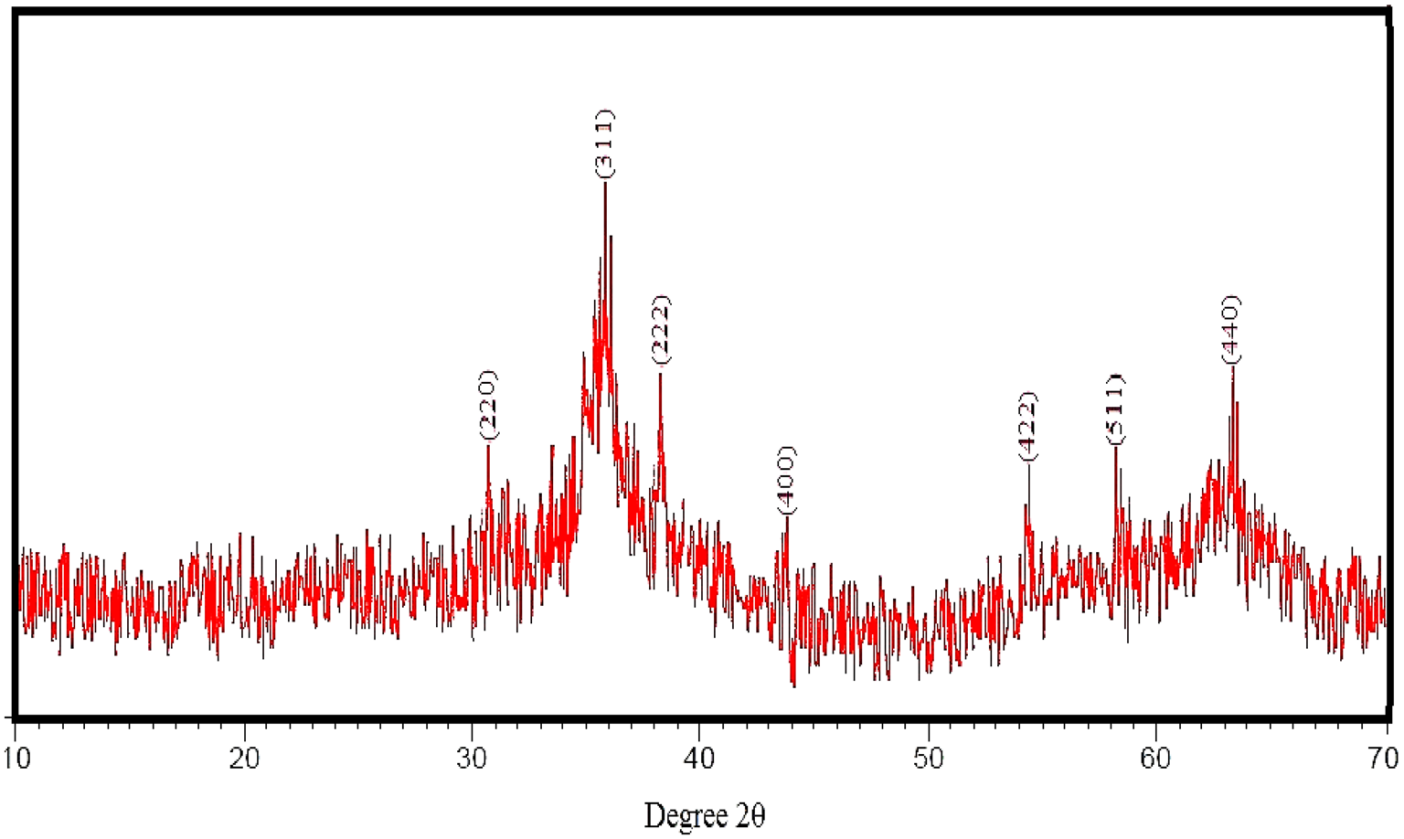
XRD pattern of the green-synthesized nickel-ferrite nanowhiskers.

The crystal and fuzzy structure of the synthesized nickel-ferrite nanostructures in the range 10–70° is shown in Fig. 1. The peaks observed at 2Ɵ correspond to the reverse spinel structure of nickel-ferrite nanostructures. The diffraction peaks correspond to lattice plane plates (220), (311), (222), (400), (422), (511), and (440)^53^.

**Figure 2:**
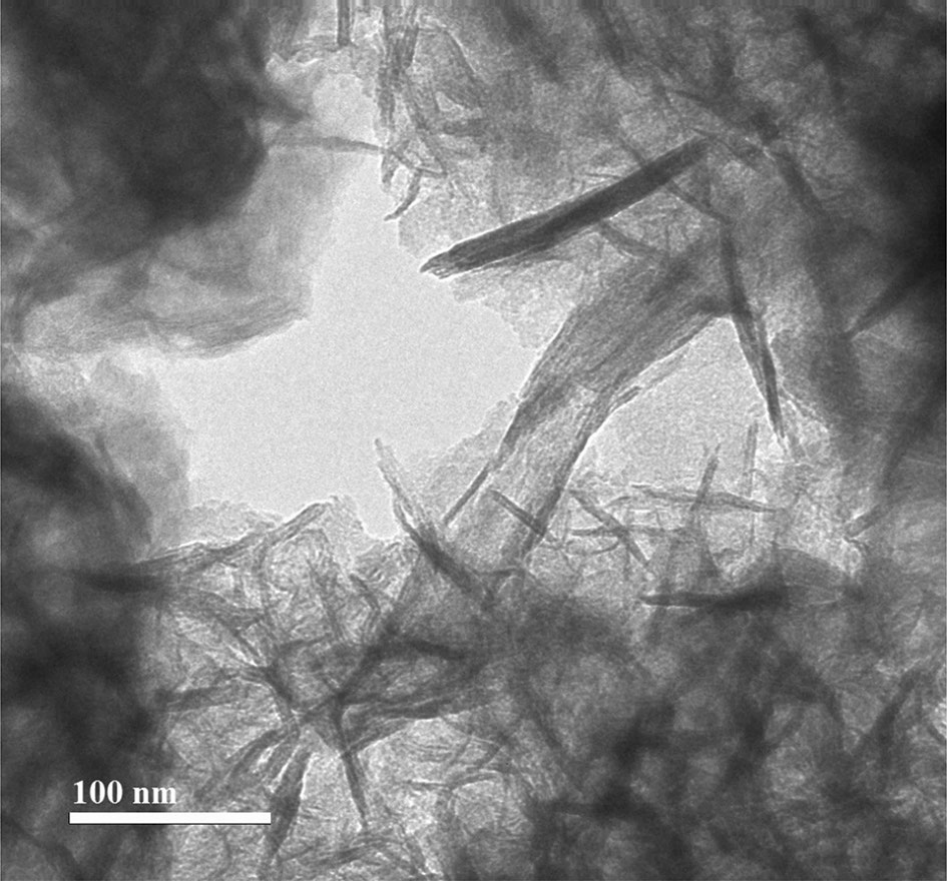
HR-TEM image of the green-synthesized nickel-ferrite nanowhiskers.

The structure of nickel-ferrite nanostructures is shown in HR-TEM image (Fig. 2, scale bar: 100 nm). According to the images, the surface of the synthesized nanostructures was smooth and even. The nanostructures were needle-like filaments (whiskers). Each hair-like strand has grown significantly in its longitudinal direction. Due to the presence of an interplate space of at least 10 nm, no aggregation or agglomeration of particles could be detected between hair-like strands such as NiFe_2_O_4_ nanowhiskers with soft surfaces. The size of these particles was less than 10 nm in width and more than 100 nm in length.

**Figure 3:**
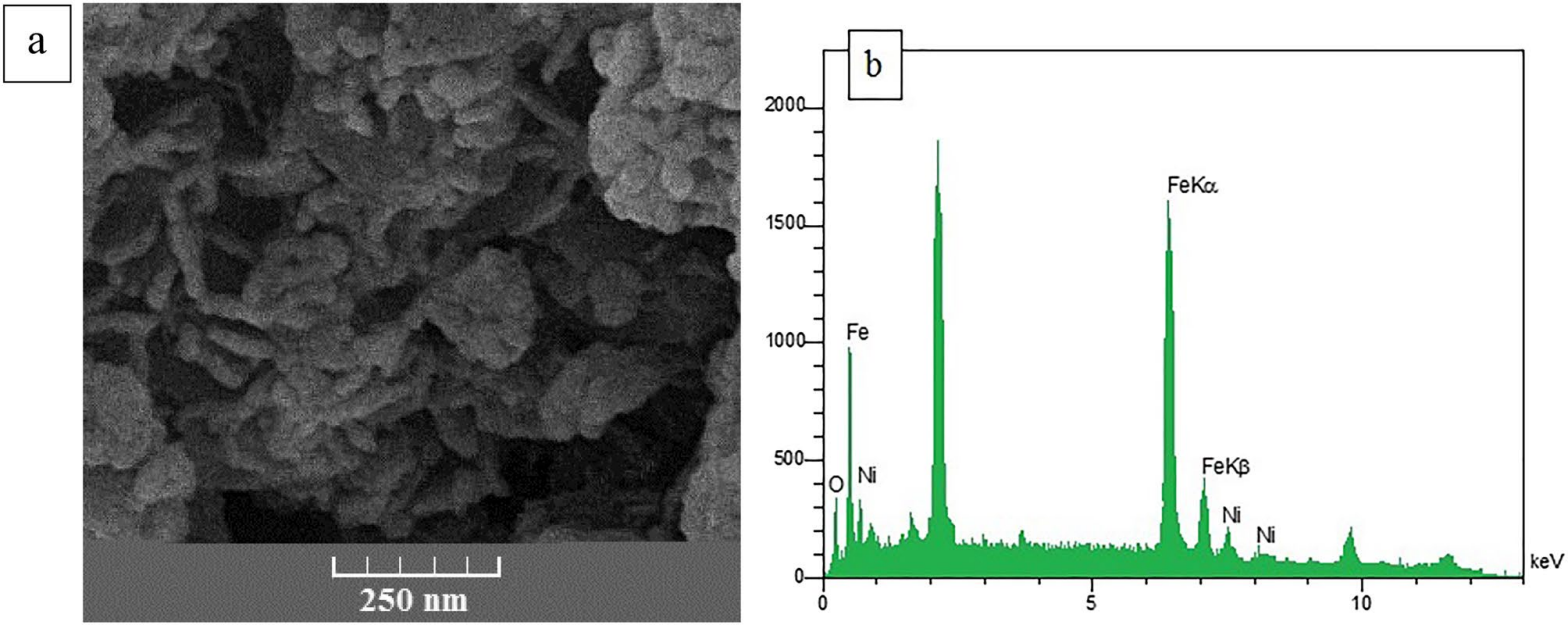
SEM and EDX images of green-synthesized nickel-ferrite nanowhiskers.

Figure 3a shows the surface morphology and number of constituents originated from NiFe_2_O_4_ nanowhiskers. As shown in SEM image, NiFe_2_O_4_ nanostructures are filamentous. The presence of nickel, oxygen and iron in the structure of the synthesized nanostructure was confirmed by EDX spectrum (Fig. 3b). EDX spectra demonstrated element ratios of Ni, Fe and O are 12, 24 and 48%, respectively.

### Efficacy evaluation of nickel-ferrite nanowhiskers on L. major

**Figure 4:**
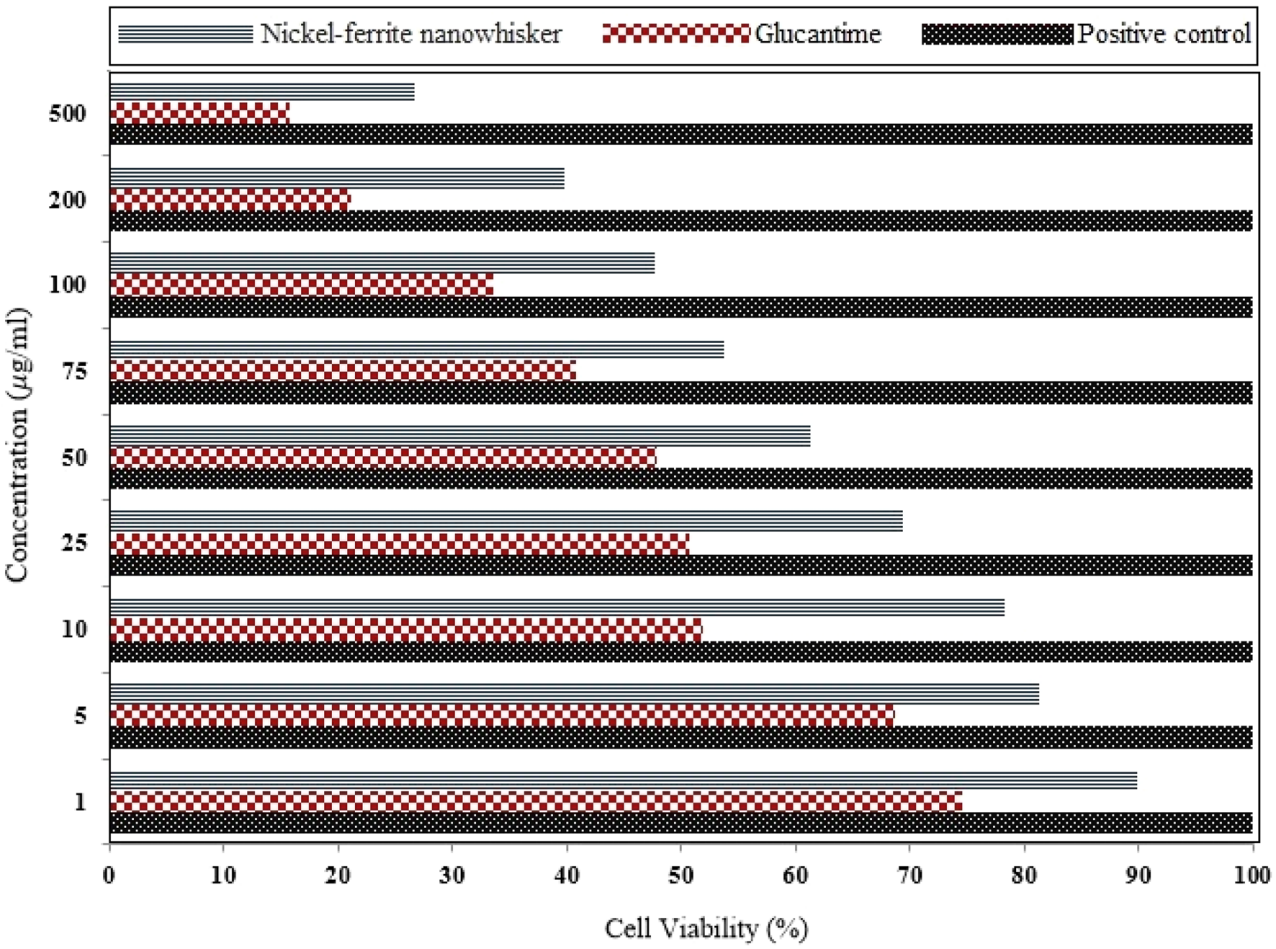
Survival percentage of *L. major* after 48 h of the exposure to different concentrations of nickel-ferrite nanowhiskers.

The effectiveness of nickel-ferrite nanowhiskers with different concentrations for 48 h was evaluated based on MTT assay against *L. major* promastigotes (Fig. 4). Based on the obtained results, the survival rate of parasitic promastigotes was considerably lowered, when the concentration of nanostructures was increased. By applying nickel-ferrite nanowhiskers (with a concentration of 500 μg/ml), the survival of promastigotes was reduced to ≃ 26%, accordingly.

Nickel-ferrite nanowhiskers in a dose-dependent manner reduced the survival rate of *L. major* promastigotes, that when compared to the control group, this difference was statistically significant (p < 0.05). In MTT assay, IC_50_ on *L. major* promastigotes was about 100 μg/ml.

## Discussion

**Figure 5:**
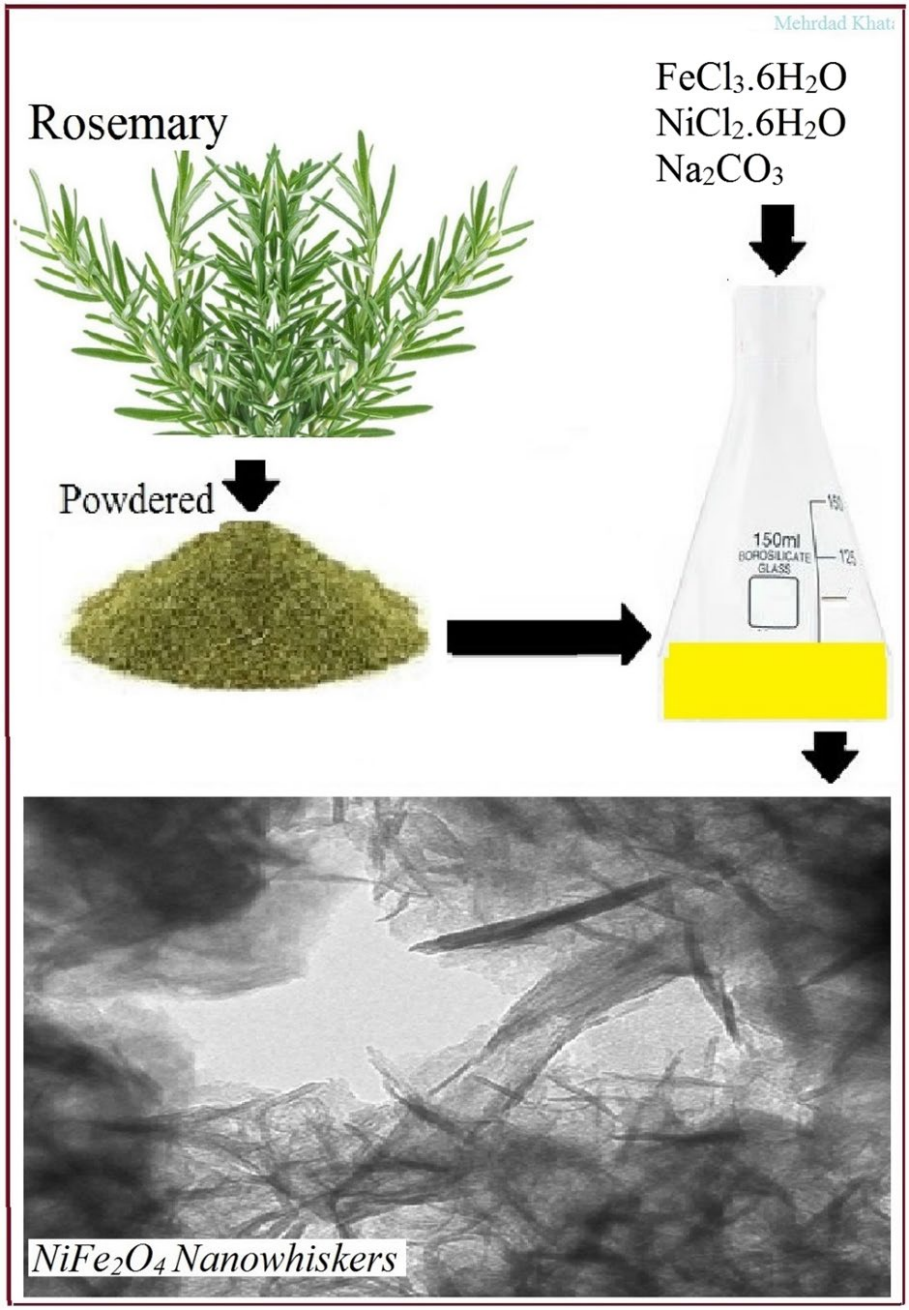
Schematic of nickel-ferrite nanowhiskers synthesis using rosemary extracts.

Nickel-ferrite nanowhiskers were synthesized in one step using rosemary phenolic extract at minimal cost (Fig. 5).

The cubic spinel structure of these spherical particles was identified by XRD analysis. Long strands of hair could be detected with a distinct morphologically margin of the whisker in HR-TEM.

Leishmaniasis is one of the most important infectious diseases in the world with 30,000 deaths annually^54^, which leads to the death by damaging macrophage cells, spleen, bone marrow, and liver. With the advancement of technology and the inadequacy of current therapeutic drugs, the application of nanostructures with unique properties and plant extracts with antimicrobial activities has recently attracted the attention of researchers^55^. Today, various nanostructures with different structures, suitable permeability, targeting properties, low toxicity, lack of resistance, high stability, and cost-effectiveness properties are widely deployed in the treatment of different diseases^54,56^. The transfer of amphotericin to glycine polymer-coated iron oxide nanoparticles could significantly reduce Leishmania parasite in the spleen^57^. Nickel oxide nanoparticles have anti-leishmaniasis activity against amastigotes and promastigotes of *Leishmania tropica* parasite^58^. But to the best of our knowledge, there are no studies about leishmanicidal activity of nickel-ferrite nanowhiskers against *L. major* promastigotes.

## Materials and methods

### Greener synthesis of NiFe_2_O_*4*_ nanowhiskers

Rosemary leaves were collected from Kerman University Garden, Kerman, Iran. The rosemary leaves were collected in accordance with applicable institutional (Kerman University), national, and international rules and legislation. It was verified by the Iranian Botanical Survey, whose voucher specimen number was 1400/1 deposited at the Department Pharmacognosy, Kerman University.

Fresh rosemary leaves were picked from the plant at the flowering stage. The leaves were disinfected using sodium hypochlorite for 3 min, and were washed 5 times with sterile deionized water and dried at 27 °C. 100 g of fresh rosemary leaves were warmed for one hour at 80 °C in 500 ml deionized water. In the next step, the mixture of leaves and deionized water was allowed to stand for one hour at room temperature and finally, filter paper was employed to separate the extract. To synthesize nickel-ferrite nanowhiskers, 1.7 g of iron (III) chloride (FeCl_3_.6H_2_O, 98%, Merck) was dissolved on a heater at a temperature of 65–70 °C with 100 ml of aqueous rosemary extract by a strainer. 0.4 g of nickel chloride (II) (NiCl_2_.6H_2_O, 99%, Merck) was added to the above mixture and dissolved at the same temperature by a strainer. Then, one molar solution of sodium carbonate (Na_2_CO_3_ anhydrous, ≥ 99.5%, Sigma-Aldrich) was added dropwise to bring the pH of the mixture to 10. Then, the mixture was stirred for about 3 h at 65–70 °C. The resulting nanostructures were separated through centrifuging, and then were washed with ethanol-deionized water and deionized water. Finally, these nanostructures were dried for 16 h at 60 degrees Celsius in an electric oven and ground into a soft powder^59^.

### Nickel-ferrite nanowhisker characterization

The crystal structure, morphology, and weight percentage of NiFe_2_O_4_ nanowhiskers were studied using XRD (X'PertPro, Panalytical; with Cu lamp), HRTEM (Sigma VP, ZEISS), and scanning electron microscope (SEM) coupled with energy-dispersive X-ray spectroscopy (EDX, TESCAN, Czech Republic).

### Toxicity evaluation of nickel-ferrite nanowhiskers against *L. major*

The Center for Disease Control, Ministry of Health, in Iran, introduced Glucantime (meglumine antimoniate) as the chosen medicine for the treatment of all clinical types of leishmaniasis. Promastigotes of *L. major* were cultivated at 24 °C in a 25 ml flask with RPMI1640 (Roswell Park Memorial Institute media), 10% fetal bovine serum (FBS), and 2% penicillin and streptomycin. A colorimetric cell viability approach was deployed to quantify viable cells in micro-well plates to examine the impact of the produced nickel-ferrite nanowhiskers on *L. major* promastigotes. Tetrazolium has a positive charge and may easily permeate living cells, converting MTT from a soluble to an insoluble dye compound that this procedure called MTT assay. In brief, 100 μl of stationary phase promastigotes (5 × 10^4^) cells/ml were introduced to a 96-well tissue culture plate. Following that, 100 μl of nickel-ferrite nanowhiskers (1, 5, 10, 25, 50, 75, 100, 200, and 500 μg/ml) were applied to each well and maintained for 48 h at 25 °C. Following the incubation period, 10 μl of MTT solution at concentration of 5 mg/ml was applied to each well and stored at 25 °C for 4 h. After that, cold isopropanol was utilized as a solvent for Formazan crystals, resulting in a purple colour. ELISA reader was employed to detect each well's absorption at 493 nm (BioTek-ELX800, Winooski, Vermont, USA). Promastigotes were cultured in drug-free RPMI1640 medium as control sample, and culture medium without promastigote and drug was also utilized as a pure sample. In SPSS software, the 50% inhibitory concentration or IC_50_ was computed for all concentrations studied using the Probit test.

## Conclusion

In this study, nickel-ferrite nanowhiskers were eco-friendly synthesized using aqueous extract of rosemary. XRD, FESEM-EDAX, and HR-TEM evaluations confirmed the spinel and needle-like structures of the prepared nanostructures. Results obtained from in vitro studies revealed that the toxicity of nickel-ferrite nanowhiskers was improved against *L. major* promastigotes by increasing the concentration of nickel-ferrite nanowhiskers and treatment duration.
